# Structure–reactivity relationships in CO_2_ hydrogenation to C_2+_ chemicals on Fe-based catalysts

**DOI:** 10.1039/d4sc06376g

**Published:** 2024-12-16

**Authors:** Jie Zhu, Shamil Shaikhutdinov, Beatriz Roldan Cuenya

**Affiliations:** a Department of Interface Science, Fritz Haber Institute of the Max Plank Society Faradayweg 4-6 14195 Berlin Germany shaikhutdinov@fhi-berlin.mpg.de

## Abstract

Catalytic conversion of carbon dioxide (CO_2_) to value-added products represents an important avenue towards achieving carbon neutrality. In this respect, iron (Fe)-based catalysts were recognized as the most promising for the production of C_2+_ chemicals *via* the CO_2_ hydrogenation reaction. However, the complex structural evolution of the Fe catalysts, especially during the reaction, presents significant challenges for establishing the structure–reactivity relationships. In this review, we provide critical analysis of recent *in situ* and *operando* studies on the transformation of Fe-based catalysts in the hydrogenation of CO_2_ to hydrocarbons and alcohols. In particular, the effects of composition, promoters, support, and particle size on reactivity; the role of the catalyst's activation procedure; and the catalyst's evolution under reaction conditions will be addressed.

## Abstract 2

This review critically discusses recent *in situ* and *operando* studies on Fe-based catalysts in CO_2_ hydrogenation, in particular focusing on the effect of composition, promoters, support and particle size.

## Graphical abstract

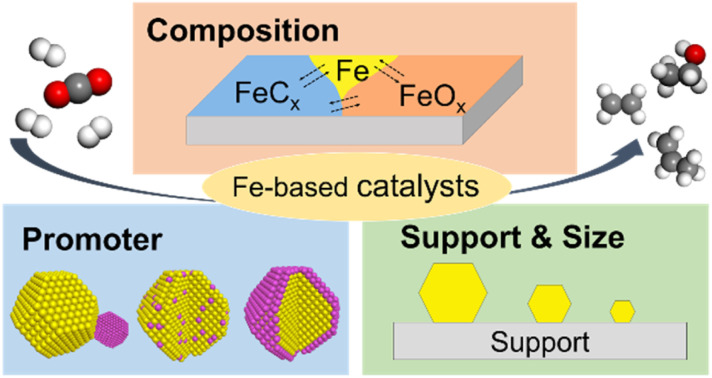

## 1. Introduction

The continuously increasing emission of carbon dioxide (CO_2_) into the Earth's atmosphere and related climate changes have given rise to enormous interest in the chemical conversion of CO_2_ as a renewable carbon source into value-added chemicals through catalytic reactions. Using “green” hydrogen, CO_2_ hydrogenation is considered to be a promising strategy to achieve a CO_2_-neutral economy.^1–3^ While considerable progress has been made in converting CO_2_ into C_1_ products such as CO,^4,5^ CH_4_ ^6,7^ and methanol,^8–10^ the production of C_2+_ chemicals (hydrocarbons and alcohols) remains highly desirable due to their broader industrial applications.^11,12^ To some extent, this latter process resembles the famous Fischer–Tropsch Synthesis (FTS) that uses syngas (CO + H_2_) as a feedstock. Moreover, the CO_2_ hydrogenation reaction to C_2+_ is often referred to as CO_2_-FTS. For the classical FTS process, the catalysts based on iron (Fe), cobalt (Co) and ruthenium (Ru) are the most efficient for carbon chain growth.^13^ However, for the hydrogenation of CO_2_, Ru- and Co-based catalysts were found to largely produce methane (CH_4_), with only limited C_2+_ production.^14–16^ On the other hand, Fe-based catalysts showed great potential for producing long-chain hydrocarbons, ranging from C_2_–C_4_ olefins to diesel-range hydrocarbons,^17–20^ and also for producing C_2+_ alcohols.^21^ A combination of the Fe catalysts with zeolite catalysts can further upgrade the product distribution through oligomerization, isomerization, and aromatization reactions.^22^ Due to the superior chain growth ability and also their low cost, Fe-based catalysts are currently considered as the most promising candidates for the production of C_2+_ chemicals *via* CO_2_ hydrogenation on an industrial scale.^18^

Structural and chemical changes, observed for Fe-based catalysts during the synthesis and the reaction itself, along with the complex reaction network, all present significant challenges for in-depth understanding of the structure–reactivity relationships for these catalysts. Typically, the catalyst synthesis starts with iron oxide as a precursor which undergoes reduction, carburization, and re-oxidation during its initial activation and reaction,^17,23,24^ often resulting in the simultaneous presence of multiple iron phases, including metallic Fe(0), and Fe(ii) and Fe(iii) oxides (FeO, Fe_3_O_4_, Fe_2_O_3_) and also carbides (Fe_3_C, Fe_5_C_2_).^25–28^ The structural dynamics of the Fe catalysts has been intensively studied in the closely related FTS process, which revealed the compositional and morphological changes, both in the bulk and at the surface.^29–33^ However, unlike FTS, where both CO and H_2_ behave as reducing agents, CO_2_ may additionally cause considerable oxidation of Fe. Surface reactions including oxygen removal, carbon deposition, carburization, oxidation, and hydrogenation become more complex. Obviously, there is a dynamic interplay between the reaction microenvironment and the surface structure of the catalyst that in turn alters surface reactions.^34^ In addition, metallic iron and iron carbide phases are sensitive to air exposure, which introduces some uncertainty in their identification. In this respect, *ex situ* studies which link the reactivity and the structural properties of a catalyst either prior to or after the catalytic tests need to be taken with certain precautions and critically analyzed, since in most cases the active sites are formed during activation or in the course of the chemical reaction. Therefore, studies on the dynamics of catalysts during the reaction become crucial for identifying the active phases/sites and for gaining a deeper understanding of the reaction mechanisms, which are pre-requisites for the rational design of more efficient and durable catalysts.^35^

**Fig. 1:**
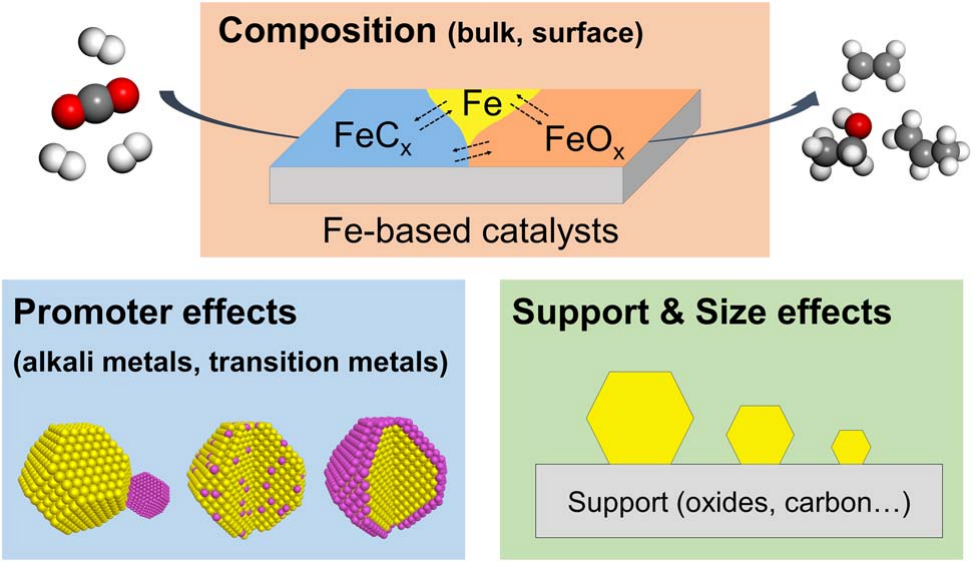
Schematic representation of several factors affecting the reactivity of Fe-based catalysts in the CO_2_ hydrogenation reaction.

In the past decade, several comprehensive reviews on CO_2_ hydrogenation to C_2+_ products have been published in the literature, focusing on catalyst structures, reaction mechanisms, and even on reactor design for various metal catalysts.^36–39^ Also, there are excellent review/perspective papers highlighting the dynamic evolution of heterogeneous catalysts in a broad range of reactions.^35,40–42^ Most recently, Ding *et al.* published an excellent review on the dynamic structure of Fe-based catalysts in CO_*x*_ hydrogenation, but mainly of CO.^33^ Thus, we are here exclusively focusing on CO_2_ hydrogenation to C_2+_ hydrocarbons and alcohols, discussing the most recent studies on the structural and chemical evolution of Fe-based catalysts. In particular, we focus on the effects of composition, promoters, support, and particle size on reactivity (Fig. 1). We also highlight the importance of *in situ* and *operando* characterization using advanced techniques described in detail in several prior reviews, including those from our own group.^43,44^ In the concluding section, we discuss the challenges and opportunities for future studies of this industrially important reaction.

## 2. Phase transformations and surface composition

**Fig. 2:**
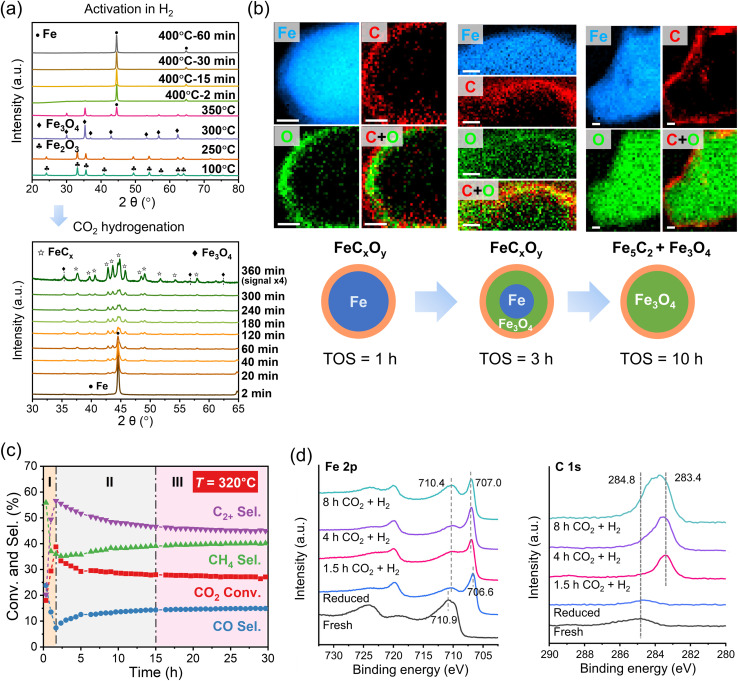
Structural evolution and catalytic performance of Fe catalysts during CO_2_ hydrogenation. (a) *In situ* XRD patterns showing the reduction of Fe_2_O_3_ to Fe in pure H_2_ (8 bar) and the phase transition during the reaction (H_2_/CO_2_ = 3; 320 °C; 8 bar). Adapted with permission from ref. 49. Copyright 2023, Elsevier. (b) Element distribution maps of spent catalysts after 1, 3 and 10 hours of reaction and (c) catalytic performance as a function of the reaction time (H_2_/CO_2_ = 3; 320 °C; 30 bar). Scale bars, 10 nm. Adapted with permission from ref. 34. Copyright 2022, The Authors, published by AAAS. (d) Quasi *in situ* Fe 2p and C 1s XPS spectra of an Fe_2_O_3_ catalyst measured after different treatments in a high-pressure cell, as indicated. Reaction conditions: H_2_/CO_2_ = 3; 300 °C; 1 bar. Adapted with permission from ref. 50. Copyright 2023, American Chemical Society.

Preparation of the iron catalysts usually starts with iron oxides such as Fe_2_O_3_ and Fe_3_O_4_, which are stable under ambient conditions. Pristine Fe-oxides in the CO_2_ hydrogenation reaction primarily yield CO and water *via* the reverse water gas shift (RWGS) reaction.^45,46^ Depending on the reaction conditions, the oxides transform into metallic Fe and iron carbide phases (FeC_*x*_) during the reaction.^47,48^ The latter shifts the product distribution towards C_2+_ hydrocarbons,^46^ suggesting that, to make the catalyst active, the oxides must be first reduced or “activated”. *In situ* X-ray diffraction (XRD) measurements revealed sequential reduction of Fe_2_O_3_ to Fe_3_O_4_ and then to Fe during heating to 400 °C in H_2_ as shown in Fig. 2a.^49^ Subsequent introduction of the reaction mixture of CO_2_ and H_2_ (1 : 3 molar ratio) at 320 °C showed fingerprints of FeC_*x*_ carbide formation within the first 20 min. After 6 h of time on stream (TOS), the Fe phase fully transformed into a mixture of Fe_5_C_2_, Fe_3_C, and Fe_3_O_4_. The spatial distribution of oxide and carbide phases was obtained by scanning transmission electron microscopy (STEM) combined with electron energy loss spectroscopy (EELS) in so-called *quasi in situ* measurements. (Henceforth, the term “*quasi in situ*” stands for the measurements on samples transferred from the reactor to the corresponding analytical tool without exposure to the ambient atmosphere.) The results showed that oxygen migrates from the surface inwards into the particle, while carbon remains at the surface (Fig. 2b).^34^ Based on additional high-resolution transmission electron microscopy (HRTEM) images, it was concluded that the initially metallic Fe particles transformed into a core–shell like structure, with the core primarily composed of Fe_3_O_4_, while the surface contained both Fe_3_O_4_ and Fe_5_C_2_, after 10 h of TOS (320 °C; 30 bar). The results also indicated that structural transformations at the surface are quite different from those in the bulk. While (bulk-sensitive) quasi *in situ* Mössbauer spectra showed a mixture of oxide and carbide phases reaching the steady state at *ca.* 3 h of TOS, the surface composition studied by quasi *in situ* X-ray photoelectron spectroscopy (XPS) showed continuous surface oxidation for more than 10 hours.^34^ Importantly, the transformation of the metallic surface into FeC_*x*_ and FeO_*x*_ is accompanied by an increase of CO_2_ conversion and C_2+_ hydrocarbon selectivity, from 18 to 39%, and from 20 to 57%, respectively (see region I in Fig. 2c). However, further surface oxidation slows down the activity (region II in Fig. 2c), indicating that excess surface FeO_*x*_ leads to catalyst deactivation.^34^

In a similar study performed at ambient pressure (1 bar), Kondratenko and co-workers^50^ using quasi *in situ* XPS showed that the surface consists of Fe(0) with small amounts of FeO_*x*_ after activation in H_2_. During CO_2_ hydrogenation, metallic Fe transformed into an FeC_*x*_ phase, which was concluded based on the small shift of the Fe 2p_3/2_ XPS peak from 706.6 to 707.0 eV and appearance of “carbidic” carbon (at 283.4 eV) in the C 1s region (Fig. 2d). *In situ* XRD showed rapid formation of Fe_5_C_2_ and Fe_3_C phases. As the reaction proceeded, the catalyst lost its activity and selectivity to hydrocarbons in favor of CO, although the surface and bulk did not undergo considerable oxidation during this period. However, *in situ* Raman in combination with C 1s XPS data indicated coke formation. By correlating the structural information with temporal analysis of H_2_ and CO_2_ activation and steady-state isotopic transient kinetic analysis (SSITKA) results, the authors came to the conclusion that coke inhibits the adsorption and activation of both CO_2_ and H_2_, and suppresses the C–C coupling reaction.^50^

Compared to the commonly studied hematite (α-Fe_2_O_3_) precursor, maghemite (γ-Fe_2_O_3_) behaves differently.^51^ During the reduction in H_2_, these two oxide phases transformed into α-Fe and γ-Fe, respectively, albeit with a portion of Fe_3_O_4_ as observed by *in situ* XRD and Raman. Interestingly, *operando* XRD measurements showed the formation of χ-Fe_5_C_2_ from α-Fe, and θ-Fe_3_C from γ-Fe phases, respectively, during the CO_2_ hydrogenation reaction (H_2_/CO_2_ = 3; 25 bar; 350 °C).^51^

Therefore, Fe carbides, which are widely recognized as the active phases for the classical FTS process,^52^ appear to be also crucial for CO_2_ hydrogenation, since the formation of FeC_*x*_ is accompanied by the increased selectivity to C_2+_ hydrocarbons. Such correlations have inspired researchers to directly synthesize FeC_*x*_ catalysts, with treatment in a CO atmosphere (so called “activation” in CO) being the most straightforward and efficient method. *In situ* XRD and Raman studies showed that Fe_2_O_3_ was first reduced to Fe and then carburized to form Fe_5_C_2_ as the temperature increased to 350 °C.^53^ The prepared Fe_5_C_2_ catalysts exhibited 54% selectivity to C_2+_ hydrocarbons and only 3% selectivity to CO. However, the Fe_5_C_2_ phase was further transformed during the reaction. *Operando* Raman spectra revealed the gradual appearance of FeO_*x*_-related bands after 60 hours on stream, and complementary XPS, XRD and Mössbauer data confirmed a partial oxidation of Fe_5_C_2_ into Fe_3_O_4_, which is accompanied by a decrease in activity.^53^ It is interesting to note a quite low selectivity to CO and substantial selectivity to CH_4_, which were observed on the pure Fe_5_C_2_ catalysts. Also, Liu *et al.* reported 50% CO_2_ conversion, with 51% of the products being C_2+_ hydrocarbons, 46% CH_4_, and the remaining 3% CO.^54^ Extrapolation of the product distribution to zero conversion led to the conclusion that RWGS and methanation are the primary reactions on pure Fe_5_C_2_, and that most C_2+_ hydrocarbons resulted from the secondary hydrogenation reaction of CO produced through the FTS route.

In addition to Fe_5_C_2_, the Fe_3_C phase is also active in the CO_2_ hydrogenation reaction. However, its role remains poorly understood even for a much more explored FT synthesis.^39,55,56^ Theoretical calculations predicted both Fe_3_C and Fe_5_C_2_ to show a lower barrier for CO_2_ dissociation and hydrogenation than metallic Fe or Fe_3_O_4_.^57^ Experimentally, it was shown that Fe_3_C exhibits a high RWGS rate at atmospheric pressure,^47,48^ while it facilitates hydrocarbon formation at elevated pressures.^58^ However, the product distribution obtained for the Fe_3_C and Fe_5_C_2_ phases seems to critically depend on the catalyst preparation and the reaction conditions used. For example, in Zhang's work,^51^ a mixture of Fe_3_O_4_ and Fe_3_C formed *in situ* from the γ-Fe_2_O_3_ precursor showed much higher selectivity towards C_5+_ hydrocarbons than a mixture of Fe_3_O_4_ and Fe_5_C_2_ formed from α-Fe_2_O_3_ (16% *vs.* 3%). In contrast, Zhu *et al.* demonstrated that the individual Fe_3_C phase exhibits a similar hydrocarbon distribution to Fe_5_C_2_ (both prepared by CO pretreatment of the α-Fe_2_O_3_ precursor), but a slightly lower CO_2_ conversion (31% *vs.* 38%).^34^ Note also that Fe_3_C may be an intermediate phase in the evolution of the iron catalyst, *i.e.*, the carburization Fe → Fe_3_C (carbon deficient) → Fe_5_C_2_ (carbon rich),^34^ and re-oxidation Fe_5_C_2_ → Fe_3_C → Fe_3_O_4_.^53^

Obviously, if not controlled, the catalyst activation in CO resulting in Fe carbide formation may additionally cause coke deposition. In the great majority of cases, there is an overlayer of carbonaceous species formed on the carbide surface which cannot be ignored when testing the catalytic performance of the “as-prepared” FeC_*x*_ catalysts. For example, this carbon overlayer can block certain active sites, thus causing an inaccurate comparison of intrinsic activity when normalized to the surface area.

The carburization and re-oxidation processes are not limited to the Fe catalysts prepared from the Fe-oxide precursors. Also, iron nitride Fe_2_N nanoparticles (NPs), despite being encapsulated by a carbon shell, were found to undergo phase transformation to Fe_5_C_2_ under CO_2_ hydrogenation conditions.^59^ XRD data showed the carburization to occur in the CO_2_ + H_2_ mixture at temperatures as high as 175 °C. *In situ* diffuse reflectance infrared Fourier transform spectroscopy (DRIFTS) results revealed the formation of Fe–NCO species, which were further hydrogenated into gas-phase NH_3_ and carbonyl iron (Fe–CO) intermediates, the latter leading to the Fe_5_C_2_ formation. The resulting catalysts showed a selectivity of 54% for C_2+_ products and 31% for C_2_–C_4_ olefins at 250 °C and 10 bar.

### Active phases

Certain correlations observed between the chemical composition of the Fe catalysts and their catalytic performance provided some rationale about the possible active phases (active sites) in this reaction. In a widely reported model, CO_2_ is first hydrogenated to CO on the Fe oxide surface *via* the RWGS reaction, and the produced CO further reacts with H_2_ on the Fe carbide to form C_2+_ chemicals through the FTS route.^22,60^ This model suggests that the Fe oxide is essential to initiate the reaction. However, the above-mentioned dynamic studies showed that a higher content of surface FeC_*x*_ (usually Fe_5_C_2_) resulted in a higher CO_2_ conversion and yield of C_2+_ hydrocarbons, while the formation of excessive FeO_*x*_ led to catalyst deactivation.^46^ These findings made researchers revisit the necessity and the role of the Fe oxide in this reaction.^61^ Indeed, there are results showing that the Fe carbide phase is also active in the RWGS reaction, even with a higher activity than that of Fe oxides.^47,48^ Moreover, both theoretical and experimental studies indicated that CO_2_ and H_2_ activation proceed more easily on the Fe carbide than oxide surfaces.^57,62,63^ Therefore, it is plausible that the sequential RWGS-FTS tandem route can, in principle, occur on the single Fe_5_C_2_ phase, *i.e.*, without invoking the FeO_*x*_ phase.^54^ Kondratenko's group also suggested the C_2+_ production on FeC_*x*_ without the formation of CO in the gas phase.^61^ These studies can rationalize the superior catalytic performance of pure Fe_5_C_2_ catalysts towards C_2+_ hydrocarbons.

It is interesting that pure FeC_*x*_ catalysts formed by activation in CO prior to the reaction showed high CH_4_ selectivity, which is at variance with the FeC_*x*_ surface formed *in situ* during the CO_2_ hydrogenation reaction over the Fe catalyst activated in H_2_. Note that adding Fe_3_O_4_ to Fe_5_C_2_ can reduce the CH_4_ selectivity.^54,64^ This could be indicative of a synergistic effect between Fe_3_O_4_ and FeC_*x*_, which can be influenced by their ratio and even spatial proximity (see more details below).^64,65^ All in all, the FeC_*x*_ carbides are considered thus far as the major active phases, with FeO_*x*_ suppressing CH_4_ production and enhancing the C_2+_ selectivity, whereas the excessive oxidation of FeC_*x*_ leads to deactivation.

### Reaction microenvironment

During the CO_2_ hydrogenation reaction, the chemical compositions of both the bulk and the catalyst surface evolve into a mixture of FeO_*x*_ and FeC_*x*_ irrespective of the initial state of the pre-catalyst. Based on classical thermodynamics, the carburization and oxidation of Fe depend on the chemical potentials of carbon (*μ*_C_) and oxygen (*μ*_O_) above the surface, which may be significantly influenced by reactants, intermediates, and products.^66^ For initially pure FeO_*x*_, the product is mainly CO and the microenvironment favors its evolution to FeC_*x*_. As the FeC_*x*_ content increases and further hydrogenation of CO proceeds, a substantial amount of H_2_O is produced, causing an increase in *μ*_O_ and hence making FeO_*x*_ thermodynamically more favorable.^34^ Overall, these two processes continuously compete with each other, altering the catalytic performance, which in turn affects the reaction microenvironment. As a result, a delicate balance between carburization and oxidation seems to exist during the reaction. Consequently, the catalyst surface may always consist of a mixture of FeO_*x*_ and FeC_*x*_. Note that metallic Fe can be oxidized by either CO_2_ or water, and obviously more water is formed in the CO_2_ hydrogenation reaction than in FTS. Thus, the substantial oxidation of the catalyst surface stands out as the significant difference between these two, FTS and CO_2_-FTS, processes. Bulk structure evolution is further influenced by factors such as kinetics and the mobility of carbon and oxygen atoms in the surface and the bulk.

The effect of water became an interesting topic that has drawn increasing attention from researchers.^67^ Co-feeding 5 vol% H_2_O significantly accelerated the surface oxidation, as found by quasi *in situ* XPS.^34^ To remove the water formed during the reaction, Chaudret *et al.* used a molecular sieve that adsorbs water.^68^ The authors observed the transformation of Fe NPs into FeC_*x*_ in a CO_2_ hydrogenation atmosphere even at 230 °C, whereas only oxidation was found at this temperature in the absence of the molecular sieve. Such an approach was even applied in reactor designs.^69^ The hydrophilic/hydrophobic properties of the catalyst surfaces may affect the interaction between water and surface iron species and hence the reaction-induced surface transformation. For instance, Xu *et al.* coated Fe–Mn catalysts with hydrophobic silane species, which reduce water retention on the catalyst surface during FTS and thereby protect iron carbides from water-induced oxidation.^70,71^ In principle, this approach is applicable to the CO_2_ hydrogenation reaction.^72^ However, a too thick hydrophobic layer may have a negative effect, *i.e.*, accelerating the oxidation of FeC_*x*_.^73^ Also, the hydrophobic carbon shell formed on the Fe carbide particles during the carburization step can minimize the water effect. In particular, alkali metal promoters, which usually enhance carbon deposition, suppress water-induced oxidation in both FTS^74^ and CO_2_-FTS (see more details below). This protective effect of carbon overlayers can explain the considerably slower oxidation of Fe_5_C_2_ particles initially prepared by CO activation,^53^ as compared to the FeC_*x*_ carbide phase formed *in situ* during the reaction.

Finally, CO_2_ hydrogenation on Fe catalysts exhibits strong pressure dependence. For example, Visconti *et al.* found that at atmospheric pressure the CO selectivity was close to 95%, but higher reaction pressures suppressed CO selectivity to 12% at 5 bar and 10% at 10 bar, thus shifting the product distribution towards C_2+_ hydrocarbons.^75^ Note that different partial pressures of products (*e.g.*, CO and H_2_O) may also lead to different degrees of carburization and oxidation at the surface. In fact, changing the reaction conditions, including temperature, pressure, feed gas composition (*e.g.*, H_2_/CO_2_ ratio) and even space velocity, can alter the reaction microenvironment and thus the surface composition of the working Fe catalysts.^34^ Therefore, the different catalytic performance may result from both the reaction conditions and the dynamic surface composition. In such a highly sensitive catalytic system, some factors are difficult to decouple, and real-time monitoring of the catalyst structure is of particular importance.

## 3. Promoter effects

**Fig. 3:**
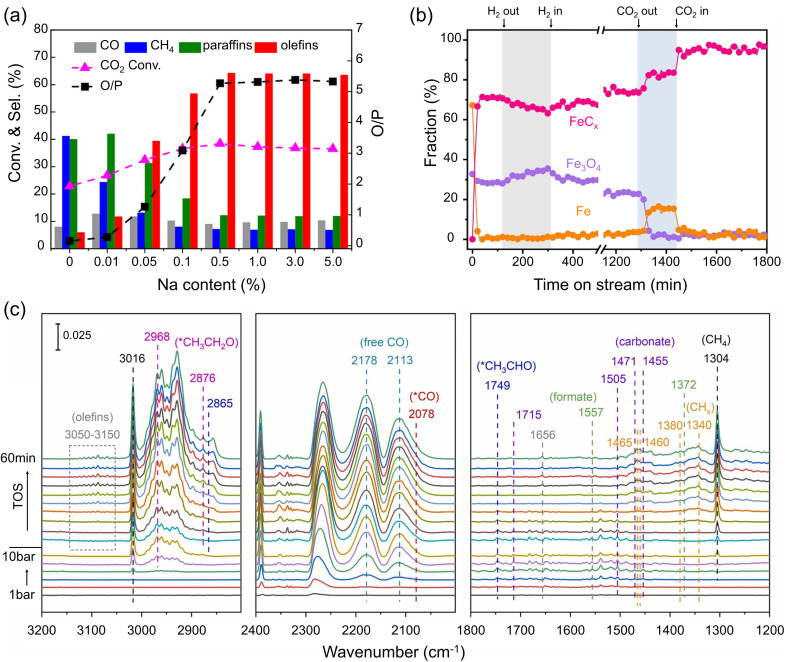
(a) Catalytic performance (conversion, selectivity, and olefin-to-paraffin ratio (O/P)) measured on Na-promoted Fe catalysts as a function of the Na content. Reaction conditions: H_2_/CO_2_ = 3; 30 bar; 320 °C. Adapted with permission from ref. 85. Copyright 2018, American Chemical Society. (b) *In situ* XRD-based fraction of the different Fe phases in the Na-promoted Fe catalysts (3 at%) during CO_2_ hydrogenation (H_2_/CO_2_ = 3; 300 °C). Arrows indicate the time when neither H_2_ nor CO_2_ was fed in the shaded area. Adapted with permission from ref. 86. Copyright 2023, Elsevier. (c) *In situ* DRIFTS spectra on an Fe catalyst promoted with Na and S. The spectra were collected while increasing the pressure from 1 bar to 10 bar and under reaction conditions (320 °C, 10 bar, H_2_/CO_2_ = 3) (from bottom to top). Adapted with permission from ref. 87. Copyright 2024, Elsevier.

Despite many efforts, pure Fe catalysts showed low selectivity to C_2+_ products. To improve the catalytic performance, alkali metals were extensively investigated as promoters in this reaction that: (i) suppresses CH_4_ formation and shifts the product distribution towards long-chain hydrocarbons, particularly to olefins; (ii) improves long-term stability.^76–83^ For example, selectivity towards C_2_–C_4_ olefins increased to 2, 22 and 27% after adding, respectively, 1, 2, and 5 wt% potassium (K) to the FeO_*x*_ precursor.^84^ As for sodium (Na)-promoted catalysts with only 0.01 wt% added, the CH_4_ selectivity decreased from 41 to 24%. Further increasing the Na content to 0.5% reduced CH_4_ selectivity to 7%, and simultaneously increased the selectivity towards total olefins from 6 to 64% (Fig. 3a).^85^ In this section, we discuss the effects of alkali metals on the nature of the Fe phases and elementary reaction steps such as adsorption, dissociation, C–C coupling, and hydrogenation.

First, alkali metals promote the chemisorption of CO_2_ and weaken that of H_2_.^79,88,89^ Li, Na, K, Rb, and Cs were found to affect the local electronic state of Fe sites in the FeC_*x*_ phase.^90^ Microkinetic analysis by temporal analysis of products (TAP) experiments suggested that CO_2_ adsorption and dissociation were enhanced by alkali metals in the order: Li < Na < K (all at 0.1 at% loading). Conversely, the ability of FeC_*x*_ to activate CO and H_2_ was hindered, and K showed a stronger effect than Li and Na. It was further proposed that the Allen scale electronegativity is a good descriptor for both activity and product selectivity.^90^ Density functional theory (DFT) calculations also suggested that the presence of K lowers the energy barriers for CO_2_ dissociation.^62^ As a consequence, even microenvironments with moderate *μ*_C_ may promote the C–C coupling process triggering the production of C_2+_ hydrocarbons. Additionally, alkali metals impede olefin adsorption, thus suppressing their subsequent hydrogenation to paraffins, overall resulting in a higher olefin-to-paraffin (O/P) ratio.^90,91^

Alkali metals facilitate the formation of FeC_*x*_ during the reaction.^49,81,84,92,93^ Apparently, the close proximity of Fe and the promoter results in a stronger effect.^79^ In addition, alkali metals inhibit the oxidation of FeC_*x*_ during the reaction,^49,85,86^ although the fundamental reasons for this effect remain unclear. Yang *et al.* used *in situ* XRD with Rietveld analysis to investigate the effect of Na under controllably varied reaction conditions.^86^ At steady state, the unpromoted catalyst consisted of FeC_*x*_ and Fe_3_O_4_. Removing H_2_ from the feed led to a decrease in FeC_*x*_ and concomitant increase in Fe_3_O_4_ content due to oxidation by CO_2_, finally resulting in reduced catalytic activity towards C_2+_ hydrocarbons. Conversely, in the absence of CO_2_, *i.e.*, in a pure H_2_ environment, both FeC_*x*_ and Fe_3_O_4_ were reduced to metallic Fe. The addition of Na stabilized the catalyst composition during these “pulse” experiments, protecting catalytically active FeC_*x*_ from oxidation and reduction (Fig. 3b), thereby enhancing its catalytic stability.^86^

The state of alkali metal species present during the reaction remains not fully understood, as they may easily interconvert during the reaction. In the “as-prepared” catalysts, K may exist as K_2_O, K_2_CO_3_, and KOH, but they become unstable at reaction temperatures. Gascon *et al.* used XPS and ^39^K nuclear magnetic resonance (NMR) spectroscopy to show that K_2_CO_3_ on the Fe catalysts evolved mainly into KOOCH, with small amounts of KHCO_3_ and K_2_CO_3_. The authors proposed that K firstly promotes the RWGS reaction: CO_2_ initially reacts with K_2_CO_3_ to form KHCO_3_, which then progressively transform into KOOCH, finally releasing CO.^93^ The produced CO can spill to neighboring Fe sites to carburize the surface to be ultimately hydrogenated into olefins *via* FTS. This mechanism explains why a carbon-containing K precursor, such as K_2_CO_3_, showed a stronger promotional effect than KCl and K_2_SO_4_.^79^

Compared to hydrocarbon production, the synthesis of C_2+_ alcohols requires not only C–C bond coupling, but also the insertion of oxygenate groups. *In situ* DRIFTS and theoretical calculations suggested that introducing a sulfur (S) promoter enhances the concentration and stability of the CO* intermediate on the surface.^21^ Given that alkali metals promote both the 
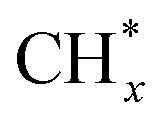
 “monomer” formation and carbon chain growth, a simultaneous use of alkali metals and sulfur as promoters may show cooperative effects on the C_2+_ alcohol production. The key is, however, to adjust the rates of C–C coupling and CO* insertion. For example, in Yao *et al.*'s study,^87^ the promotional effect of Li (0.3 wt%) on C–C coupling was rather limited, which only resulted in a slight increase in methanol selectivity when the Fe catalyst was modified with Li and S promoters. Conversely, the K promoter (3 wt%) showed a much stronger effect on C–C coupling, leading to increased selectivity towards C_5+_ hydrocarbons (42%) and C_2+_ alcohols (8%). A moderate promotional effect was observed on the Na and S-promoted catalyst at similar loadings, where the catalyst showed a CO_2_ conversion of 32% and 16% selectivity to C_2+_ alcohols.^21,87^*In situ* DRIFTS showed that in the presence of Na and S, both carbonate and formate species appeared upon exposure to the CO_2_ + H_2_ reaction mixture. Under reaction conditions, *CO, alkyl species, *CH_3_CHO and CH_3_CH_2_O* species appeared sequentially as the reaction proceeded (see Fig. 3c), pointing to the coupling reaction between alkyl and *CO ad-species. DFT calculations demonstrated that a delicate balance between the rates of dissociative and non-dissociative CO adsorption must have been achieved in these experiments.

In summary, alkali metals, particularly potassium and sodium, can modulate the reaction microenvironment by increasing CO_2_ adsorption and dissociation while weakening H_2_ adsorption, promote the formation of FeC_*x*_ and prevent its excessive oxidation during the reaction, improving both activity and stability towards C_2+_ production. Thanks to their ability to tune the coupling of 
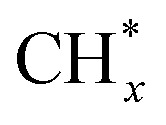
 species, a moderate combination with promoters like sulfur, which stabilize CO* intermediates, can achieve the production of C_2+_ alcohols (see more details below).

## 4. Support effects

For catalytic reactions using precious or noble metals, it is common to use oxide supports to increase metal dispersion (to reduce the cost) and also to prevent thermally- or reaction-induced metal sintering. In the case of 3d-metal catalysts, in particular iron oxides, which are one of the most abundant compounds on the Earth, there is no real reason to use a support in its classical meaning, unless the oxide behaves as a structural promoter, primarily to increase the specific surface area of the active phase. In addition, a support is unavoidable for catalytic studies aimed at examining the size effect on reactivity, in particular for NPs in the sub-nanometer range, which would otherwise be impossible to stabilize against sintering at catalytically relevant temperatures.

The results obtained for oxide-supported Fe-based catalysts in the CO_2_ hydrogenation reaction indicate that the support can considerably influence the catalytic performance.^81,94–96^ The supports may affect the chemical state of Fe during both activation and reaction. For example, FeO as an intermediate phase was observed during activation in CO on a catalyst supported on a monoclinic (m-) ZrO_2_, but not on a catalyst supported on tetragonal (t-) ZrO_2_.^95^ Moreover, less coke was formed on the former catalyst as monitored by *in situ* XRD and Raman spectroscopy. Consequently, m-ZrO_2_-supported K-promoted Fe catalysts exhibited 39% CO_2_ conversion and a high selectivity towards C_2_–C_4_ olefins (43% among all hydrocarbons). The morphology of the nanocrystalline support also affected the reduction of Fe-oxides. For example, CeO_2_ nanocubes exposing (100)-oriented facets were found to facilitate the reduction, as compared to CeO_2_ nanorods primarily exposing the (110) planes. Using the latter support resulted in catalysts showing a higher olefin/paraffin ratio.^97^

Alumina (Al_2_O_3_) is widely used as a support, and its interaction with Fe can regulate the chemical compositions of the catalyst surface. Increasing the calcination temperature of Na-promoted FeO_*x*_–Al_2_O_3_ pre-catalysts causes a stronger interaction, hindering the reduction and carburization of the Fe-oxide.^98^ The catalysts pre-calcined in air at 900 °C contained 25% Fe_5_C_2_ after the CO activation step, while those calcined at 350 °C showed a higher degree of carburization, resulting in 50% Fe_5_C_2_ and 13% Fe_7_C_3_. Correlation between the catalytic performance and surface composition, together with *in situ* DRIFTS and DFT calculations, demonstrated that a higher content of surface FeC_*x*_ leads to a higher CO_2_ conversion, and a higher proportion of Fe_5_C_2_ in the carbide phase results in a higher chain growth possibility.

**Fig. 4:**
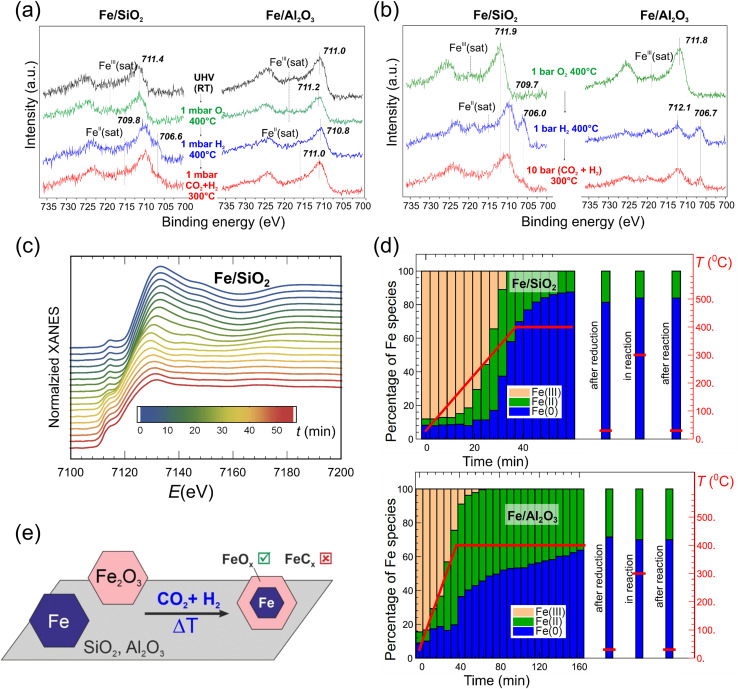
(a) Fe 2p region of the NAP-XPS spectra and (b) quasi *in situ* XPS spectra of model catalysts, prepared on SiO_2_/Si(001) and Al_2_O_3_(0001) substrates using polymer-free Fe-oxide micelles (4 nm in size). The NAP conditions (in a) and *ex situ* treatments (in b) are indicated. (c) *In situ* Fe K-edge XANES spectra of nanocrystalline (powder) SiO_2_-supported Fe-oxide catalysts, prepared using the same micelles as for the model catalysts (a) and (b), during heating to 400 °C in H_2_. (d) Fraction of different Fe species, obtained by linear combination analysis of XANES spectra (top, Fe/SiO_2_; bottom, Fe/Al_2_O_3_), during reduction in H_2_, under reaction conditions (10 bar; H_2_/CO_2_ = 3; 300 °C), and after cooling to room temperature. (e) Schematic representation of the structural evolution of the nano-sized Fe catalysts. Adapted with permission from ref. 99. Copyright 2021, American Chemical Society.

It should be noted that small Fe NPs, especially those smaller than 10 nm, behave quite differently during the reaction (see more details in Section 5). The effect of the oxide support on the surface and bulk evolution of such small NPs was investigated by Luna *et al.*^99^ FeO_*x*_ NPs with a narrow size distribution around 4 nm were prepared by an inverse micelle encapsulation method. The micelles were deposited on nanocrystalline SiO_2_ and Al_2_O_3_ supports for *in situ* X-ray absorption spectroscopy (XAS) studies, and also on SiO_2_/Si(001) and Al_2_O_3_(0001) substrates for model studies using near ambient pressure (NAP)-XPS. The NAP-XPS spectra (Fig. 4a) showed that Fe(iii) was reduced to Fe(ii) and partially to Fe on a model Fe/SiO_2_ catalyst upon activation at 400 °C in 1 mbar H_2_, with Fe being re-oxidized during the CO_2_ hydrogenation at total 1 mbar pressure at 300 °C. In contrast, the Fe/Al_2_O_3_ model catalyst remained mainly in the Fe(iii) state after both activation and reaction. Moreover, the state of Fe formed during the reaction was independent of the initial state of the pre-catalyst, *i.e.*, Fe oxide or pure metallic Fe NPs prepared on both supports by physical vapor deposition (PVD). Quasi *in situ* XPS measurements performed after reduction at a catalytically relevant pressure (1 bar) revealed a higher degree of Fe reduction on the Al_2_O_3_-supported NPs as compared to SiO_2_ (Fig. 4b). After the CO_2_ hydrogenation reaction at 10 bar, the surface was found to be re-oxidized, with Fe(ii) and Fe(iii) species dominating the XPS spectra, independently of the oxide supports.

A complementary *in situ* Fe K-edge X-ray absorption near edge structure (XANES) study showed that the fraction of metallic Fe species in the Fe/Al_2_O_3_ catalyst was significantly lower than in Fe/SiO_2_ (65 and 85%, respectively), indicating that FeO_*x*_ NPs on Al_2_O_3_ are more resistant to reduction. Nonetheless, the state of Fe formed by the H_2_ activation step remained unchanged during the reaction (Fig. 4c and d), *i.e.*, in contrast to the XPS results clearly showing surface re-oxidation in the reaction atmosphere. The main findings obtained by bulk-sensitive XAS and surface-sensitive XPS, namely, a core (metal-rich)-shell (oxide-rich) structure, are schematically depicted in Fig. 4e. Interestingly, there were no signs of Fe carbide formation during the reaction in XAS and XPS measurements on these nano-particulate catalysts, which produced light hydrocarbons, with the O/P ratio being considerably affected by the nature of the oxide support used. Still, it remains to be studied whether these findings can be assigned to pure support effects or whether they are also affected by the nano-sized nature of the active phase.

Compared to oxide supports, carbon supports were thought to exhibit a weaker interaction with Fe oxide. On the other hand, a carbon support may serve as a source of carbon for Fe carbide formation. Using *in situ* XANES spectroscopy, Muhler and co-workers found that SiO_2_-supported FeO_*x*_ NPs can only be reduced to Fe(ii) in H_2_ at 380 °C, while NPs supported on nitrogen-doped carbon nanotubes (CNTs) underwent full reduction to the metallic state.^100^ Consequently, the lower activity and C_2+_ selectivity of the Fe/SiO_2_ catalysts were attributed to the strong iron-silica interaction, which prevents reduction and hence carburization of Fe. In another study, Wu *et al.* prepared Fe/C catalysts using honeycomb-structured graphene as the support and potassium as the promoter, which showed 59% selectivity towards C_2_–C_4_ olefins, stable during 120 hours on stream.^80^ The long-term stability was attributed to the confinement effect of the porous structure of the support, which prevented the sintering of FeC_*x*_ NPs during the reaction. Indeed, the mean size of the FeC_*x*_ particles only slightly increased from 14 nm after 24 hours to 16 nm after 120 hours on stream.

In principle, the support not only influences and stabilizes the particular state of iron, but can directly participate in the reaction through the interaction with gas molecules and spillover-based mechanisms. For example, acid sites on the amorphous alumina support can promote the oligomerization of olefins first produced on the FeC_*x*_ sites, as shown by *in situ* DRIFTS.^24^ Too strong acidity led to the pyrolysis of long-chain hydrocarbons, while moderate acidity in the Fe/AlO_*x*_ catalysts showed a high selectivity (52%) to linear α-olefins (78% in C_4+_ olefins) that was stable for 450 h of TOS.^24^ For the case of a single-wall CNT support, those with a large curvature facilitated the dissociation of C–O bonds, thus promoting the formation of 
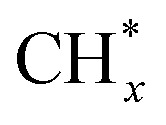
 monomers. Additionally, the confined space in CNTs can serve as a “nano-reactor”, where the residence time of light olefins can be longer, thus providing the possibility for oligomerization reactions and for achieving a high C_5+_ hydrocarbon selectivity, up to 40%.^101^

Therefore, for supported Fe catalysts, not only the textural properties of a support (*e.g.*, morphology, pore structure, specific surface area), but also their surface properties, such as acidity and hydrophilicity,^73,102^ play a significant role in the catalyst evolution and the surface reactions.

## 5. Size effects

Similar to many reactions on metal catalysts, CO_2_ hydrogenation is also quite sensitive to the metal particle size. In case of Ru,^103^ Rh,^104^ Ir^105^ and Ni^106^ catalysts, large particles favored CH_4_ formation, whereas reducing the NP size down to single atoms shifted the product distribution towards CO. The reactivity of Fe-based catalysts also showed size dependence, albeit being more complex because of a relatively large variety of products. It should be mentioned that sometimes the particle size referred to the size of Fe particles in the “as-prepared” (*i.e.*, Fe-oxide) catalyst, or “activated” (reduced), or even spent catalyst. The latter constitutes a problem in the field, since the structure of these catalysts, including their size, likely changes during the reaction, leading to questionable size–reactivity correlations. The problem is especially drastic for single-atom pre-catalysts, where C–C coupling products might be assigned to the concomitant presence of small clusters or nanoparticles formed during operation.

**Fig. 5:**
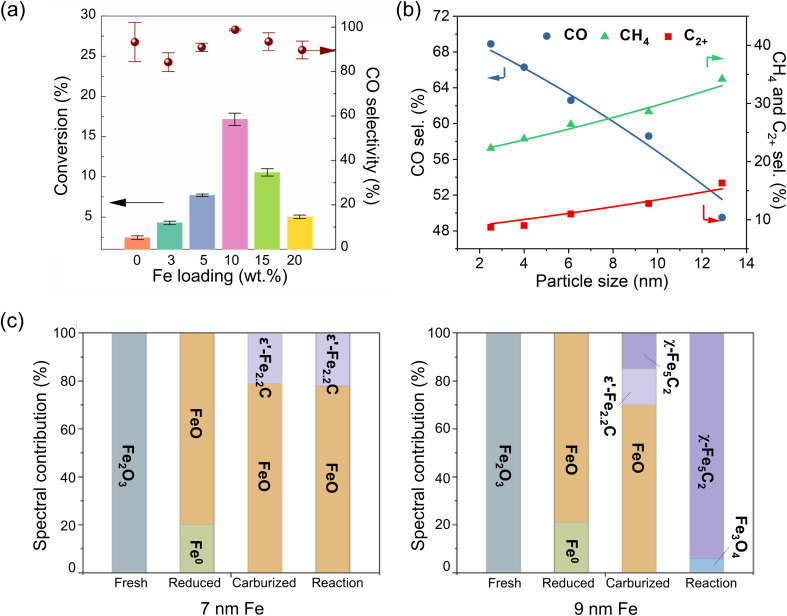
(a) Catalytic performance of Fe catalysts supported on MoS_2_ as a function of Fe loading (in wt%). Reaction conditions: 10 bar; 300 °C; H_2_/CO_2_ = 3. Adapted with permission from ref. 107. Copyright 2021, American Chemical Society. (b) Product selectivity as a function of Fe particle size on H_2_-activated Fe/ZrO_2_ catalysts. Reaction conditions: 30 bar; 320 °C, H_2_/CO_2_ = 3. (NB: The space velocity was adjusted for each catalyst to reach a similar CO_2_ conversion (∼13%)). Adapted with permission from ref. 45. Copyright 2020, American Chemical Society. (c) Composition of fresh, reduced, carburized, and post-reaction Fe catalysts determined by quasi *in situ* Mössbauer spectroscopy for two different initial particle sizes. Adapted with permission from ref. 108. Copyright 2024, Elsevier.

Based on the extended X-ray absorption fine structure (EXAFS) results of MoS_2_-supported Fe catalysts, Zheng *et al.* concluded that Fe was present primarily as single atoms even in the highly loaded catalysts, up to 10 wt%.^107^ The catalysts reduced in H_2_ showed 100% CO selectivity at 300 °C at atmospheric pressure. Increasing the pressure to 10 bar only led to the formation of small amounts of CH_4_ (<2%) and traces of C_2_ and C_3_ hydrocarbons, with CO dominating the product distribution (Fig. 5a). Note that close to 100% selectivity to CO remained for more than 80 hours, and no Fe–Fe bonds were found in EXAFS spectra measured on the 10 wt% Fe/MoS_2_ catalyst after reaction. CO_2_ conversion increased as the Fe loading increased from 3 to 10 wt%, presumably due to the higher density of the Fe single atoms. However, further increase of the Fe loading to 15 and 20 wt% resulted in decreased CO_2_ conversion due to the formation of Fe clusters, although CO was the main product.

Leybo *et al.* synthesized Fe phthalocyanine-derived single-atom catalysts supported on boron nitride.^109^ Again, the “as-prepared” catalysts exhibited 100% selectivity towards CO at 20 bar and 200–230 °C. However, as the reaction temperature increased to 320 °C, the product distribution shifted towards CH_4_ (15%) and C_2+_ hydrocarbons (10%). Interestingly, the latter products were observed even at lower reaction temperatures, if the catalyst was pre-reduced in H_2_ at 350 °C prior to the reaction. Based on a TEM study, the effect was explained by the formation of small Fe NPs (∼3 nm) at elevated temperatures, either during the reduction step or under reaction conditions. Therefore, the observed Fe sintering largely eliminates the initial difference in particle size.

This general trend that larger Fe NPs favor hydrocarbon production was further proven by Xie *et al.* who used Al_2_O_3_ supports with different pore sizes to prepare Fe_2_O_3_ particles ranging from 5 to 23 nm.^110^ The selectivity to C_2+_ and C_5+_ hydrocarbons showed a volcano-type relationship with respect to the initial particle size, with a maximum C_2+_ selectivity achieved at around 5–8 nm. Note, however, that the alumina supports were synthesized by quite distinct methods, so the results obtained may be influenced by both, size and support, effects.

Zhu *et al.* prepared a series of ZrO_2_-supported Fe catalysts with particle sizes in the reduced catalysts varying from 3 to 13 nm, as determined by a number of techniques such as CO chemisorption, XRD and TEM.^45^ As the particle size increased, selectivity to C_2+_ hydrocarbons and CH_4_ continuously increased from 9 to 16% and 22 to 34%, respectively, while that of CO decreased from 69 to 50% (Fig. 5b). Interestingly, the authors observed that the CO_2_ conversion and C_2+_ selectivity increase with TOS on the smallest 3 nm NPs, and attributed this behavior to the size effect *via* reaction-induced sintering. Kondratenko's group examined unsupported Fe_2_O_3_ NPs of larger sizes, *i.e.* 15–30 nm. In this study, smaller particles, possessing more defects, were found to facilitate the reduction and formation of defective Fe_5_C_2_ NPs, which showed enhanced CO_2_ and CO adsorption.^111^

One reason for the particle size effect is that small Fe NPs/clusters and single atoms often exhibit non-metallic properties. When supported, they may be harder to reduce because of their strong interaction with the underlying support.^112,113^ A lower degree of reduction is not conducive to the *in situ* formation of active FeC_*x*_.^111,114^ According to *in situ* XRD results, the reduction of ZrO_2_-supported FeO_*x*_ particles starts at a lower temperature for 13 nm NPs, as compared to 6 nm NPs, and the formation of FeC_*x*_ during CO_2_ hydrogenation proceeds much faster.^45^ In another case of carbon-supported K-promoted catalysts,^108^ the Fe_2_O_3_ NPs showed a similar degree of reduction to FeO in H_2_ at 400 °C for two samples with 7 and 9 nm initial average particle size (Fig. 5c). However, during the activation in the mixture of H_2_ and CO at 280 °C, these two samples showed considerably different compositions. The “7 nm” sample contained 21% of Fe_2.2_C, while the “9 nm” sample had 15% of Fe_5_C_2_ and 15% of Fe_2.2_C. More significantly, after the CO_2_ hydrogenation reaction (300 °C, 11 bar), the “9 nm” sample became almost fully carburized (85% Fe_5_C_2_) while the “7 nm” sample showed no changes.

The coordination of the Fe atoms at the particle surface may also play a role. Indeed, DRIFTS spectra of CO, used as a probe molecule, showed that the ratio of bridged and linear CO adsorption sites increased as the particle size increased from 3 to 13 nm, indicating a higher fraction of low-coordinated Fe sites on the smallest Fe particles.^45^ Since the carbon chain growth requires a close proximity of 
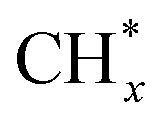
 “monomers”, the C–C coupling reaction becomes more favorable on the well-ordered facets dominating on the largest particles.

In summary, the particle size effects on reactivity may originate from both electronic and geometric effects, although the predominance of one *versus* the other is strongly linked to the nanoparticle/cluster size range considered, with electronic effects becoming most relevant for sizes in the sub-nanometer range. So far, the activity of catalysts containing single Fe atoms and small clusters in C_2+_ production has been very low, most likely because of: (i) the complex C–C coupling reactions requiring more than a single site; and (ii) the low degree of Fe reduction for the sub-nm particles and hence the limited formation of the FeC_*x*_ carbide phase due to their strong interaction with the support. Nonetheless, a single-atom catalyst can serve as a “pre-catalyst” for preparation of catalysts with a narrow particle size distribution. The optimal particle size of Fe in the CO_2_-FT reaction seems to be in the range of 10–15 nm.

## 6. Bimetallic Fe-based catalysts

Adding a second metal (such as a 3d transition metal or noble metal) to Fe is an effective strategy to improve the selectivity and catalytic stability of the Fe catalysts.^15,25,115,116^ Several studies have shown that easily reducible metals, such as Pt,^47^ Pd^117^ and Cu,^118^ promote the reduction of the Fe-oxide through facile H_2_ dissociation on these metals and subsequent hydrogen spillover onto the Fe-oxide surface, thereby promoting the formation of the FeC_*x*_ carbide phase under reaction conditions.

**Fig. 6:**
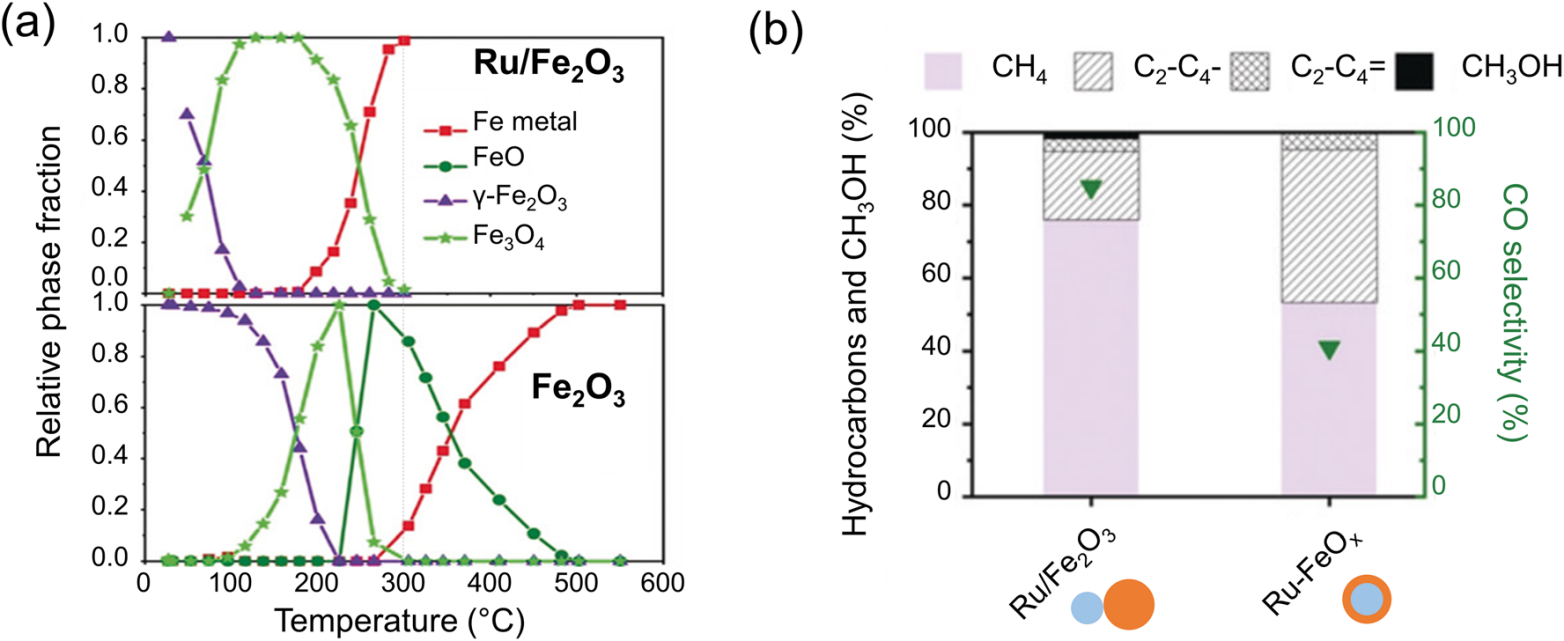
(a) Compositional changes monitored by *in situ* XANES in the Fe_2_O_3_ and Ru–Fe_2_O_3_ catalysts during heating in H_2_. (b) Catalytic performance of Ru–Fe catalysts with different Fe contents that form 4 nm- and 1 nm-thick Fe shells during the reaction. Reaction conditions: 300 °C, 6 bar, H_2_/CO_2_ = 3. Adapted with permission from ref. 119. Copyright 2019, John Wiley and Sons.

For example, Cargnello's group prepared colloidal particles in order to provide a close contact between the Ru and Fe precursors, and the particles were deposited onto the γ-Al_2_O_3_ support (with a total metal loading of 1 wt%).^119^ After calcination at 700 °C to remove organic ligands, Ru was partially oxidized and Fe was in the form of γ-Fe_2_O_3_. Based on *in situ* XAS results in a H_2_ environment, upon the complete reduction of Ru, the Fe_2_O_3_ phase was fully reduced to metallic Fe at ∼300 °C, whereas the Ru-free, reference Fe_2_O_3_ catalyst underwent a much slower transition from Fe_3_O_4_ to FeO, with no complete reduction to Fe being observed until 500 °C (Fig. 6a). *In situ* Fe K-edge XANES spectra indicated that the Ru–Fe catalyst predominantly consisted of metallic Fe and FeC_*x*_ during the reaction, with no observable contribution from FeO_*x*_. Interestingly, STEM images of the spent catalyst combined with energy dispersive spectroscopy (EDS) showed the formation of “core–shell” particles having a metallic Ru core and an FeO_*x*_ shell about 4 nm in thickness. (In fact, the shell was composed of Fe and FeC_*x*_ under reaction conditions, but was oxidized during the sample transfer through air.)

*In situ* EXAFS results revealed Ru–Fe bond formation at the interface between the Ru core and the Fe shell. The authors proposed that a relatively thick Fe shell in these particles obscured the electronic effect of Ru, and the difference in the catalytic performances of these two catalysts, *i.e.*, with and without Ru, largely stems from the different degrees of reduction of the Fe phase. Indeed, when the Ru-free catalyst was reduced in H_2_ at 550 °C, it showed similar selectivity to the Ru-promoted catalyst, where Fe was fully reduced at 300 °C. In order to prepare the catalyst, with the surface exposing more Fe atoms in direct contact with Ru, the authors synthesized Ru particles covered by a thinner Fe shell (∼1 nm), and this catalyst showed a 4-fold increase in the hydrocarbon yield (Fig. 6b), implying the strong electronic effect of Ru on the reactivity in such hetero-structures.^119^

For the Pd-promoted catalysts, *in situ* XRD showed the formation of a Pd–Fe alloy during activation in H_2_.^120^ The catalyst underwent complete Fe carburization during the CO_2_ hydrogenation reaction, in contrast to the physically mixed Pd–Fe_2_O_3_ catalyst under the same reaction conditions. Since the latter does not form the Pd–Fe alloy in the reduction step, it is the alloy formation that promotes the formation of Fe_5_C_2_ in the reaction atmosphere. Based on the DRIFTS results, the alloy phase was proposed to be responsible for the RWGS reaction and CO non-dissociative activation, while Fe_5_C_2_ is responsible for the chain growth. The reaction at the PdFe/Fe_5_C_2_ interface seems to enhance the production of C_2+_ alcohols, achieving 27% selectivity at 300 °C and 50 bar.^120^

Copper (Cu) also improves the reducibility of FeO_*x*_, and hence facilitates the formation of FeC_*x*_,^25^ and also enhances the adsorption of CO_2_ and H_2_.^115,121^ Compared to the K-promoted FeCu/Al_2_O_3_ catalyst prepared by sequential impregnation, the catalyst prepared by co-impregnation of Cu and Fe precursors exhibited a strong interaction between Fe and Cu and showed a promotional effect, with selectivity to C_5+_ hydrocarbons increasing from 10 to 14%.^122^ In a similar Fe–Cu–K–Al system, Jun *et al.* used XRD, XPS and XAS to demonstrate that K promotes Cu incorporation into the lattice of either metallic Fe or Fe carbide phases during the reaction.^123^ The synergistic effect of Cu and K led to a C_5+_ yield of 18% compared to 13% obtained on the Cu-free, Fe–K catalyst.

Fe–Cu binary oxides have emerged as superior precursors for preparing effective catalysts. Comparative studies of CuFeO_2_ delafossite, CuFe_2_O_4_ spinel, and physically mixed Fe and Cu oxides showed that the fraction of C_5+_ in all hydrocarbons produced at 300 °C and 10 bar (with ∼30% CO selectivity) increases in the order CuO–Fe_2_O_3_ (3%) < CuFe_2_O_4_ (11%) < CuFeO_2_ (66%).^25^ Note, however, that the CuFeO_2_ catalyst contained traces of Na (0.03%). For a similar CuFeO_2_ catalyst, Li *et al.* reported 67% of C_4+_ olefins (44% of CO excluded) even at ambient pressure and 320 °C.^124^

Nonetheless, among the 3d transition metals, cobalt (Co) stands out as one of the most extensively studied,^15,116,125–128^ owing to its wide application in the conventional FTS process, where metallic Co showed a much higher chain growth factor than the Fe-based catalysts, and as such it is largely used to produce heavy hydrocarbons. However, in the CO_2_ hydrogenation reaction, pure Co showed high CH_4_ formation, with only limited C_2+_ production, and was therefore used primarily as the methanation catalyst. Studies on the Fe–Co catalysts showed that the spatial distance of Fe and Co significantly influences their catalytic behavior. When two phases are well separated, the CO_2_ hydrogenation reaction occurs independently on each component, resulting in substantial CH_4_ formation on the Co sites. In contrast, intimate contact or even close proximity between Fe and Co allows the CO formed on the Fe sites (*via* the RWGS reaction) to spill over to the Co sites, which enhances the chain growth in the FTS step and promotes heavy hydrocarbon production.

Jiang *et al.* addressed the role of the inter-particle distance between Fe and Co by employing different preparation methods, including co-impregnation and physical mixing.^129^ When Fe and Co were co-impregnated on a SiC support promoted by K, the selectivity towards C_2+_ hydrocarbons increased from 38 to 57%, and the CO_2_ conversion increased from 17 to 30%, compared to the Co-free Fe catalyst. However, the physically mixed FeK/SiC and Co/SiC catalyst, *i.e.*, with a much larger inter-particle distance, mainly produced CH_4_ (79% selectivity), while it is only 3% on the Co-free Fe–K catalyst.

**Fig. 7:**
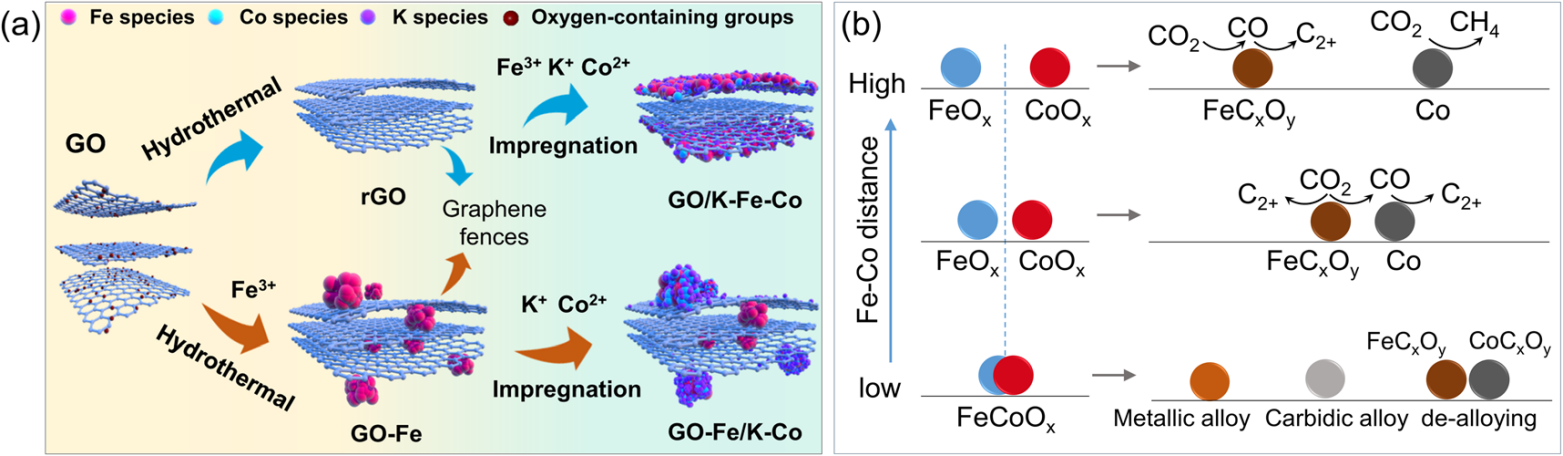
(a) Scheme showing different approaches for the synthesis of spatially distributed Fe and Co on a graphene oxide (GO) support. Reproduced with permission from ref. 130. Copyright 2024, The Authors, published by Springer Nature. (b) Schematic diagram of alloying and de-alloying behaviors of Fe–Co bimetallic catalysts during activation and the CO_2_ hydrogenation reaction.

To controllably tune the proximity of Fe and Co phases, Tsubaki's group used graphene oxide as a “fence” to separate Fe and Co precursors (Fig. 7a).^130^ For Fe and Co to be in direct contact, all precursors of Fe, Co, and K were impregnated and uniformly dispersed on the exterior surface of the graphene. When the Fe precursor was first introduced for the hydrothermal treatment of graphene, Fe was found both on the graphene surface and between the graphene layers (intercalated). Finally, Co and K were impregnated onto the exterior graphene layers. Spatial distribution was analyzed using scanning electron microscopy (SEM) with EDS mapping. Using in addition *in situ* XRD, EXAFS and XPS, the authors showed that the catalysts consisted of Fe_5_C_2_ and metallic Co under reaction conditions. In comparison to the reference Fe catalysts, which exhibited 31% selectivity to C_2_–C_4_ olefins, the Fe–Co sites formed by direct contact revealed a higher (*i.e.*, 50%) selectivity. Conversely, the spatially separated Fe–Co NPs produced almost no C_2_–C_4_ olefins, but achieved 44% selectivity to C_3_–C_4_ paraffins. It was proposed that the individual Co NPs enhance the secondary hydrogenation reactions of olefins produced on the Fe_5_C_2_ phase.

When Fe and Co precursors form a single compound, such as CoFe_2_O_4_ and Fe–Co layered double hydroxide (LDH), then reduction in H_2_ results in Fe–Co alloying.^131–135^ However, further evolution of the alloy during CO_2_ hydrogenation strongly depends on the Fe/Co atomic ratio. To recall, for individual Fe and Co catalysts, metallic Fe transforms into the FeC_*x*_ phase, while Co predominantly remains metallic, although there is some probability of Co-carbide formation. Accordingly, for an Fe-rich FeCo alloy, it mostly transforms into FeC_*x*_, with Co incorporated into its lattice, thus forming an “Fe–Co carbidic alloy”.

Kim *et al.* performed *in situ* XRD studies of a Na-promoted CoFe_2_O_4_ catalyst supported on CNTs.^134^ After reduction in H_2_, XRD showed diffraction patterns of an Fe–Co alloy. Due to the pressure limitation of their XRD setup, to simulate partial pressure of CO under realistic CO_2_ hydrogenation reaction conditions, the authors used pure CO at atmospheric pressure to treat the H_2_-activated catalyst. Only the FeC_*x*_ phase was observed, with no signature of pure metallic Co.^134^ Based on theoretical considerations, the authors inferred the formation of (Fe_1−*x*_Co_*x*_)_5_C_2_ carbide, where *x* is lower than 0.2, and even predicted its crystal structure, although direct experimental proof of the proposed structure is still missing. Nonetheless, implementation of this carbidic alloy into the fitting model showed consistency with Liu *et al.*‘s XRD, XAS and Mössbauer results, indirectly validating the carbidic alloy formation.^132^ Liu *et al.* also pointed out that when the Co/Fe molar ratio exceeds 0.5, the formation of the alloy carbide is suppressed, and that of the Co_2_C phase becomes favorable. Nonetheless, the formation of the Fe–Co carbidic alloy enhanced the production of low-carbon (C_2_–C_4_) olefins.^15,132^

At higher Co/Fe ratios, the alloy remains in the metallic state during the reaction. For example, a Co-rich Co_7_Fe_3_ alloy was formed after activation in H_2_.^136^ After reaction at 200 °C, both XRD and EXAFS revealed that the bulk composition remained as Co_7_Fe_3_, and quasi *in situ* XPS showed that both Fe and Co at the surface are in the metallic states. No carbides were observed, either in the bulk or at the surface. Theoretical calculations suggested that the Co-rich alloy is the active phase in the C–C coupling reaction between surface carbonaceous species. In contrast to the carbidic alloy that favored C_2_–C_4_ olefin production, the Co_7_Fe_3_ metallic alloy exhibited a high selectivity (63%) to jet-fuel-range (C_8_–C_16_) hydrocarbons at 10% CO_2_ conversion.^136^

De-alloying may also occur during the reaction, leading to the formation of separate phases of FeC_*x*_ and Co (or CoC_*x*_).^137^ Chen *et al.* synthesized Fe–Co alloy catalysts by ball milling of a physically mixed Fe, Co_3_O_4_ and K_2_CO_3_ powder.^137^ After 6 hours of milling treatment, XRD patterns showed the catalysts consisting of 20% Fe–Co alloy, with the rest being CoO_*x*_ and Fe. After the subsequent CO_2_ hydrogenation reaction, the fraction of Fe–Co alloy decreased to about 10%, with the major phases being Fe_5_C_2_ and Co_2_C, indicating alloy segregation. DRIFTS spectra complemented with DFT calculations suggested that CO_2_ is initially hydrogenated to CO on the Fe–Co alloy surface, which then reacted with surface carbon species on both iron and cobalt carbides for the C–C coupling step.

Several possible scenarios of Fe–Co catalyst evolution are depicted in Fig. 7b. They may additionally be affected by the proximity effects and by Fe/Co ratios, as well as the activation and reaction conditions. The bimetallic catalysts can form alloys, segregated phases, or a mixture of both.^127^ It appears that a moderate Fe–Co distance allows the rates of the RWGS reaction, methanation, 
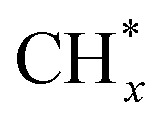
 coupling and the secondary hydrogenation of olefins to be balanced, and thereby the distribution of hydrocarbon products to be tuned.

Metal oxides can also act as electronic or structural promoters.^138–140^ For instance, both MnO_*x*_ and Na facilitated the carburization of Fe during the reaction. However, quasi *in situ* structural characterization showed that simultaneous modification with Na and Mn weakened the Fe–Mn interaction and decreased the content of the formed Fe_5_C_2_ as compared to the Na-free Fe–Mn catalyst, while MnO_*x*_ itself was transformed into MnCO_3_ under reaction conditions.^23^ On the basis of reaction kinetics analysis, it was concluded that Na and Mn-promotion of Fe catalysts allows the reaction rates of RWGS and FTS steps to be matched, and thus results in an enhanced overall reactivity and olefin selectivity. As for the ZnO promoter, ZnFe_2_O_4_ spinel is normally used as the catalyst precursor.^141,142^ During activation in CO, it first separates into ZnO and FeO phases, and the latter transforms into Fe_5_C_2_.^142,143^ Both ZnO and Na promoters stabilize Fe_5_C_2_ against over-oxidation during the subsequent reaction, as shown by *in situ* XRD, Raman and NAP-XPS.^143,144^ The *in situ* formed interface between ZnO and Fe_5_C_2_ seems to be responsible for the enhanced production of light olefins.

Bimetallic Fe-based catalysts showed higher potential for C_2+_ alcohol production as compared to monometallic Fe catalysts. FeC_*x*_ is effective for the formation and coupling of 
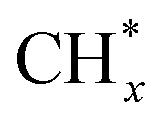
 monomers. However, to produce C_2+_ alcohol, an additional component is needed for the formation of oxygenate intermediates such as CO* or CHO*. Too strong chain growth ability leads to the production of solely C_2+_ hydrocarbons, as shown in the above-mentioned studies on Fe–Cu and Fe–Zn catalysts. Thus, to improve the selectivity to C_2+_ alcohols, one needs to balance the rate of chain growth and oxygenate insertion, which necessitates proper modification of the catalysts and optimization of the reaction conditions. For example, an amorphous ZrO_2_ support facilitated non-dissociative CO adsorption on Fe–Cu–K catalysts, resulting in a C_2+_ alcohol selectivity of 28% and CO_2_ conversion of 31% at 320 °C and 50 bar.^145^ A carbon-supported, Na-promoted Fe–Zn catalyst evolved into a ternary ZnO_*x*_–Fe_5_C_2_–Fe_3_O_4_ compound during the reaction, as shown by *in situ* XRD and quasi *in situ* XPS.^146^ It was proposed that ZnO donates electrons to the Fe sites and to the carbon support, thereby strengthening the adsorption of CO. Consequently, the catalyst exhibited 19% selectivity to ethanol at a CO_2_ conversion of 34% at 320 °C and 50 bar, with no deactivation over more than 500 hours on stream. *In situ* DRIFTS confirmed the CO-insertion mechanism for ethanol production. In addition to CO*, CHO* intermediates can also be produced on the Zn-containing phase, *i.e.*, ZnFe_2_O_4_.^147^ The interface between ZnFe_2_O_4_ and Fe_5_C_2_ on the optimized catalyst boosted the production of C_2+_ alcohols, with a proportion of 16% for all the hydrocarbon and oxygenate products at 300 °C and 50 bar. Of all alcohols produced, 98% were C_2+_ alcohols and more than 40% were C_3+_ alcohols.

To sum up, the bimetallic Fe-based catalysts offer a promising approach to improve the catalytic performance for the production of both C_2+_ hydrocarbons and alcohols. A combination of Fe with a more easily reducible metal considerably facilitates the reduction of the Fe-oxide precursor and promotes FeC_*x*_ formation. The spatial proximity and the molar ratio of the two metal precursors significantly influence alloying/de-alloying processes during the catalyst preparation, activation, and reaction. Certainly, the effects of the promoter, support, and particle size, observed for monometallic Fe catalysts, become more complex for the bimetallic systems.

## 7. Outlook

Over the past few decades, enormous efforts have been devoted to establishing structure-reactivity relationships for Fe-based catalysts in CO_2_ hydrogenation to C_2+_ chemicals. Given the complex and sensitive structural evolution, there remain some challenges and promising opportunities for future research.

### Controllable synthesis of iron carbide catalysts

Compared to χ-Fe_5_C_2_ and θ-Fe_3_C, other iron carbides like ε-Fe_2_C and Fe_7_C_3_, which showed superior performance in the FTS process,^148,149^ are less investigated in CO_2_ hydrogenation. Meanwhile, the poorly defined structure of the FeC_*x*_ phases in the existing studies on CO_2_ hydrogenation renders determination of their intrinsic activity rather difficult. It is therefore essential to synthesize single-phase iron carbide catalysts for further fundamental studies of reactions at their surfaces. Control of the elemental composition of the FeC_*x*_ phase, its crystal structure, particle size, and shape in Fe-carbide synthesis remains a significant challenge. The same applied also to their subsequent phase stabilization during the reaction. Preparation approaches like wet chemical synthesis have been developed,^150^ but the instability of iron carbides upon air exposure can readily cause surface restructuring during sample transfer. In this respect, vacuum based thin-film technologies can be a good option.^151–153^ For example, atomically defined FeC_*x*_ films were recently prepared by ethylene decomposition over the Fe thin films grown on an Au(111) substrate.^151,152^ These films can serve not only as model catalysts for fundamental studies, but also as a prototype for monolith-type catalysts suited for industrial applications. Here, a scalable industrial technology already exists for the preparation of thin film catalysts, namely that employed for solar cells (PVD) that can be easily adapted for reproducible catalyst synthesis.

The controllable synthesis of iron carbides can provide the opportunity to investigate also the shape effect in this reaction. Shape selectivity has recently been investigated for CO_2_ hydrogenation to methanol over ZnO-supported Cu_2_O nanocubes exposing solely the (001) facets.^154^ To date, the shape effect on the reactivity of Fe-carbides in CO_2_-FTS is primarily studied through theoretical simulations. For instance, Nie *et al.* found that the χ-Fe_5_C_2_(510) surface exhibits higher activity for the direct dissociation of CO_2_ into CO* and O*, while the (111) surface is more favorable for CO_2_ hydrogenation into the HCO* intermediate.^155^ Despite the different reaction pathways, both the (510) and (111) surfaces appear to be better candidates for C_2+_ hydrocarbon production as compared to (100), (11−1), (110) and (10−1) surfaces. Experimental efforts to control the initial shape of the Fe catalysts are currently limited to Fe-oxide precursors, but the question still remains on how to retain such shape under the reaction environment. The latter might be achieved by careful selection of the appropriate underlying support and carefully controlled treatments. Chen *et al.* synthesized α-Fe_2_O_3_ nanodisks of certain thickness and diameter which were enclosed with (0001) basal facets and (11−20) side facets and applied them for CO_2_ hydrogenation.^156^ However, the post-reacted catalysts displayed a quite rough surface, cracks and severe sintering, although the disk shape remained at a large scale. Very recently, Wu *et al.* reported synthesis of χ-Fe_5_C_2_ nanoparticles with specifically exposed surfaces using the conformal reconstruction of well-defined Fe_3_O_4_ nanocrystals during pre-reduction in H_2_ and activation in syngas.^157^ In fact, the prepared particles showed an Fe_3_O_4_ core/χ-Fe_5_C_2_ shell structure, with the χ-Fe_5_C_2_(202) surface being formed on Fe_3_O_4_ nanocubes exposing the (400)-oriented facets, while Fe_3_O_4_ octahedra primarily exposing the (111) facets favored the formation of the χ-Fe_5_C_2_(112) surface. This preparation allowed a look into the facet effects on reactivity of the Fe_5_C_2_ carbide in FTS. We believe that such an approach can also be applied to the CO_2_ hydrogenation reaction.

### *Operando* characterization under catalytically relevant conditions

Given the dynamic nature of this catalytic system, the real-time analysis of the catalyst structure at different time and length scales from the atomic level, meso and microscale becomes crucial. Bulk-sensitive techniques such as XRD, Raman, and XAS are currently well-suited for *operando* studies. Future efforts should be focused on improving the detection sensitivity, time and spatial resolution. For example, accurate identification of the atomic structure of the above-mentioned Fe–Co alloy catalyst is not trivial due to the naturally close similarities in the structural and electronic parameters of neighboring Fe and Co in the periodic table. Moreover, spectroscopic ensemble-averaging techniques might miss key spatially separated changes in the catalyst structure and composition, such as different oxidation states or carbide phases at different locations within the same sample or even within different regions of the same large nanoparticle. This can for instance apply to nanoparticles of a different size or those located on support regions of different characteristics or specific defects within a sample with heterogeneous characteristics either in the as-prepared state, or during reaction (changes in particle size and phase composition).^158^ Such complexity is starting to be addressed in the catalysis community by combining multi-technique ensemble-averaging characterization approaches with locally resolved spectro-microscopy methods, including synchrotron-based transmission X-ray microscopy or low energy electron microscopy combined with X-ray photoemission electron microscopy among others.

Moreover, the surface structure of the working catalyst remains poorly understood. Atomic-level understanding can, in principle, be obtained on the basis of “surface science” studies of model systems using a large variety of surface sensitive techniques. For example, Guo *et al.* visualized in real time the chain growth process during ethylene polymerization monitored by scanning tunneling microscopy (STM) on a carburized Fe(110) single crystal surface.^159^ Nevertheless, it is in many cases still unclear whether such model systems are really representative of all or at least some of the key characteristics of the real industrial catalyst, and thus, bridging the materials gap still remains a challenge. Another question to address in this community is the possible relevance of the pressure gap that most traditional surface science experiments inherently suffer from. Nilsson's group's work has recently resulted in a major leap forward in this direction by developing an advanced NAP-XPS setup enabling *in situ* measurements at pressures up to 500 mbar,^160^ whereas conventional setups mostly operate at pressures in the 1–10 mbar range. In particular, this group has investigated the surface evolution of the Fe(110) single crystal surface during CO_2_ hydrogenation. As the reaction temperature increases, no carbide formation was observed due to the very low CO concentration formed *via* the RWGS reaction on the low-surface-area single crystal catalyst. However, adding CO to the feed gas resulted in carburization of the Fe surface. Moreover, it seems possible to discriminate octahedral and trigonal prismatic carbides formed at elevated reaction temperatures.

Still, CO_2_ hydrogenation is known to be highly sensitive to the reaction pressure, where the product distribution shifts from over 90% CO at atmospheric pressure to hydrocarbons at higher pressures.^75^ Therefore, vacuum-based *in situ* characterization (at NAP conditions) needs to be complemented with quasi *in situ* measurements after high-pressure treatments to address the “pressure gap”.

### Theoretical simulation of catalyst dynamics

Theoretical calculations provide fundamental insights into the reaction mechanism at the atomic level, including the determination and distinction of reaction intermediates from spectator species, both of which would be detected experimentally and at times wrongly assigned. Nevertheless, the empirically derived ideal and still mostly “static” model for the catalyst structure and reaction microenvironment may not accurately reflect the real situation under the working conditions. The complexity in phase transition, surface reconstruction, and the interplay between the catalyst surface structure and gas-phase environment are usually underestimated. Therefore, theoretical simulations on catalyst dynamics and the corresponded reaction environment are essential for rationally establishing structure–reactivity relationships,^161,162^ which can be achieved by combining DFT, Monte Carlo-based approaches, first-principles thermodynamics and microkinetic simulations. In this respect, machine learning could be particularly effective for such complex systems.

## 8. Synopsis

It is generally accepted in the catalysis community that rational design of an efficient catalyst relies on precise structure–reactivity relationships which must be established for the catalyst in its working state, through comprehensive *in situ*/*operando* characterization. This situation holds true also for Fe-based catalysts in CO_2_ hydrogenation to C_2+_ chemicals, where considerable structural and chemical changes take place both in the bulk and at the surface during the reaction. This structural evolution may be affected by the reaction microenvironment formed above the catalyst surface, which also changes as the reaction proceeds. Nonetheless, fine-tuning the composition, as well as electronic and geometric structure, of the Fe-based pre-catalysts can serve to influence their subsequent transformation during the reaction. In particular, it has been shown to offer opportunities to balance multiple elementary reactions, including CO_2_ dissociation, chain growth, and its termination, oxygenate insertion, secondary hydrogenation and oligomerization of olefins, thereby directing the production of desired C_2+_ products.

For synthesis of C_2+_ hydrocarbons from CO_2_ hydrogenation, FeC_*x*_ carbides are believed to be the essential phases, with a certain amount of FeO_*x*_ phase improving the C_2+_ selectivity, and excessive oxidation of FeC_*x*_ leading to catalyst deactivation. Alkali metals promote FeC_*x*_ formation and prevent its over-oxidation during the reaction, significantly enhancing C_2+_ production. Both the support and size of Fe NPs affect the reducibility of the Fe-oxide precursor to metallic Fe, which is, in turn, a pre-requisite for Fe-carbide formation. Apparently, a particle size in the range of 10–15 nm is optimal for C_2+_ hydrocarbon production. In bimetallic systems, the second metal primarily facilitates Fe reduction. However, the ratio and proximity of the second metal to Fe both influence the alloying/de-alloying behavior and hence the reaction pathway. For C_2+_ alcohol production, FeC_*x*_ is responsible for 
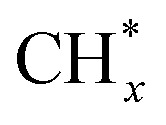
 monomer formation and chain growth, cooperatively working with another active component responsible for the insertion of oxygenate groups. Promoters such as S, Cu, Pd, and ZnO can be efficient for this purpose.

Given the dynamic nature and complexity of this catalytic system, it is crucial to establish the role of the promoter, the possible effects of size, support and shape (affecting the particle-support contact area) on reactivity, and formation of the “real catalyst” from the pre-catalyst upon activation, and its further evolution under reaction conditions. We hope that this review of *in situ*/*operando* studies aids in providing a better understanding of the Fe-based catalysts “at work”, and provides insights into active phase(s) of the catalysts ultimately resulting in the production of C_2+_ hydrocarbons and alcohols from CO_2_.

## Data availability

This study was carried out using publicly available data from the references cited.

## Author contributions

J. Z. drafted the manuscript and prepared all figures. S. S. and B. R. C. conceptualized the work and edited the final manuscript. All authors approved the manuscript.

## Conflicts of interest

There are no conflicts to declare.
